# Two Trypanocidal Dipeptides from the Roots of *Zapoteca portoricensis* (Fabaceae)

**DOI:** 10.3390/molecules19055470

**Published:** 2014-04-25

**Authors:** Ngozi Justina Nwodo, Festus Basden C. Okoye, Daowan Lai, Abdessamad Debbab, Reto Brun, Peter Proksch

**Affiliations:** 1 Department of Pharmaceutical and Medicinal Chemistry, University of Nigeria, 410001 Nsukka, Enugu State, Nigeria; 2 Department of Pharmaceutical and Medicinal Chemistry, Nnamdi Azikiwe University, 420281 Awka, Anambra State, Nigeria; E-Mail: basdenc@yahoo.com; 3 Institute for Pharmaceutical Biology and Biotechnology, Heinrich-Heine-University, D-40225 Dusseldorf, Germany; E-Mails: Laidaowan123@gmail.com (D.L.); abdessamad.debbab@uni-dusseldorf.de (A.D.); Proksch@uni-dusseldorf.de (P.P.); 4 Department of Medical Parasitology and Infection Biology, Swiss Tropical and Public Health Insitute, CH-4002 Basel, Switzerland; E-Mail: reto.brun@unibas.ch; 5 Department of Medical Parasitology, University of Basel, CH-4003 Basel, Switzerland

**Keywords:** *Zapoteca portoricensis*, dipeptides, isolation, fabaceae, trypanocidal acitivity

## Abstract

*Zapoteca portoricensis* (Jacq) HM Hernández is used with remarkable efficacy in ethnomedicinal management of tonsillitis in the Eastern part of Nigeria. Previous pharmacological studies have validated the antiinflammatory and antimicrobial activities of the crude extract. In this study, two dipeptides, saropeptate (aurantiamide acetate) and anabellamide, were isolated from the methanol root extract of *Zapoteca portoricensis* and their chemical structures deduced by one dimensional and two dimensional NMR and mass spectrometry. These compounds were isolated for the first time from this plant, and no report has been found on their previous isolation from the genus *Zapoteca*. Evaluation of their trypanocidal activity showed that compound **1** exhibited potent activity against *Trypanosoma brucei rhodesiense* with an IC_50_ value of 3.63 µM and selectivity index of 25.3.

## 1. Introduction

The genus *Zapoteca* which belongs to the family of *Fabaceae* is a glabrous seasonal shrub with slender unarmed branches. The plant is a native of West Africa, the West Indies and the Atlantic Coast of America. It is used in folk medicine in various countries for the treatment of toothache, tonsilitis, in wound healing, against diarrhoea, and as an anticonvulsant and antispasmodic. Our previous studies showed that the crude methanolic extract of the root and some of its fractions had significant anti-inflammatory, antimicrobial and antitrypanosomal properties [1,2,3]. The anti-ulcer, antifungal and antibacterial potentials have been equally documented [4,5,6].

Our literature survey showed that the chemistry of *Zapoteca portorticensis* has been poorly investigated. To date, there is no report on the isolation and characterization of any chemical constituents from this plant. We therefore decided to investigate the methanol root extract leading to the isolation of two dipeptides. Peptides generally possess unique pharmacological properties which could be exploited for therapeutic purposes. There is a growing interest in peptide therapeutics in the pharmaceutical industry based on increasing concerns about small molecular drugs and the increased attention to foods and nutraceuticals possessing health preventive or health promoting properties [7,8]. An estimated 60 peptide drugs were approved in 2010 alone, which generated annual sales of around US$ 13 billion. Dipeptides are usually metabolic products of bacteria and fungi but are only rarely isolated from plants. We report in this paper the isolation and structure elucidation of dipeptides from *Zapoteca portoricensis*, which is the first report of dipeptides from this genus.

## 2. Results and Discussion

**Figure 1:**
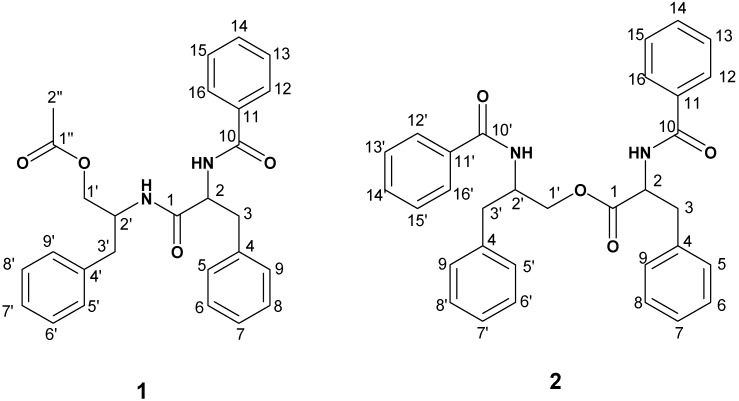
Chemical structures of the isolated compounds.

The powdered roots of *Z. portoricensis* were extracted with methanol and the crude methanol extract was successively partitioned into hexane, ethyl acetate, butanol and water fractions. The chromatographic separations of the ethyl acetate fraction, using vacuum liquid chromatography (VLC) on silica gel, Sephadex LH-20 and further purification with reverse phase semi-preparative HPLC yielded compounds **1** and **2** (Figure 1). Their structures were elucidated using ^1^H, ^13^C-NMR, DEPT, HMBC, HSQC and LC-MS spectral analyses and comparison of the data with those reported in the literature [9,10,11].

**Figure 2:**
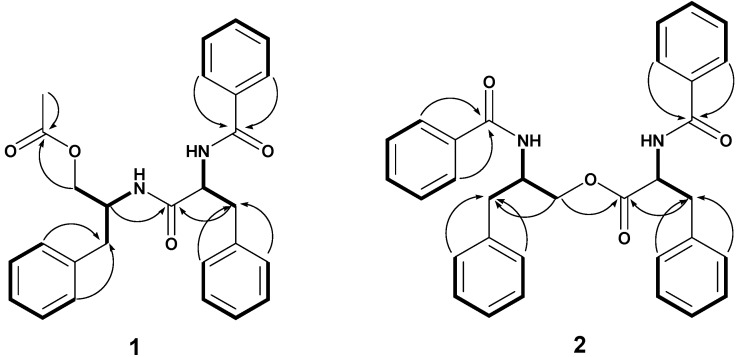
Key COSY and HMBC correlations. The thick lines show COSY correlations while the arrows show HMBC correlations.

Compound **1** was isolated as white crystals. The ESI-MS showed peaks at at *m/z* 444.20 [M+H]^+^ and 467 [M+Na]^+^ which is consistent with a molar mass of 443 and a molecular formula C_27_H_28_N_2_O_4_. The ^1^H-NMR and ^1^H^1^H-COSY of **1** exhibited two spin systems in the aliphatic region. One began with a quartet at *δ*_H_ 4.74 (1H, q) assigned to H-2 which was coupled H-3b at *δ*_H_ 3.19 (1H, dd, *J* = 5.9, 14.0) and also H-3a at *δ*_H_ 3.04 (1H, dd, *J* = 8.5, 14.0). The other spin system began with a multiplet at *δ*_H_ 4.33 (1H, m) assigned to H-2', which was coupled with H-3' at *δ*_H_ 2.73 (2H, t, *J* = 7.1). H-2' was also coupled with H-1'b *δ*_H_ 3.90 (1H, dd, *J* = 4.9, 11.3) and H-1'a at *δ*_H_ 3.79 (1H, dd, *J* = 4.2, 11.3). Two amide signals were observed at *δ*_H_ 6.72 (1H, d, *J* = 8.4, N-Hb) which showed COSY correlations with H-2 (*δ*_H_ 4.74 q) and at *δ*_H_ 5.89 (1H, d, *J* = 9.4, N-Ha) which showed COSY correlation with H-2' (4.33 m) (Figure 2). The ^1^H-NMR spectrum of **1** also showed three sets of aromatic signals. These include signals *δ*_H_ 7.20–7.28, which integrated to five protons assigned to H-5, H-6, H-7, H-8 and H-9. Another set of aromatic signals were observed at *δ*_H_ 7.10–7.18 which integrated to three protons assigned to H-6', H-7' and H-8' together with a duplet at *δ*_H_ 7.05 (2H) assigned to H-5'/H-9'. Finally the ^1^H-NMR spectrum displayed aromatic spin signals of AA'BB'C type at *δ*_H_ 7.69 (2H, d, *J* = 7.2) assigned to H-12/H-16; *δ*_H_ 7.42 (2H, t, *J* = 7.6) assigned to H-13/H-15 and *δ*_H_ 7.50 (1H, t, *J* = 7.4) assigned to H-14. Compound **1** was identified as saropeptide (2-(2-benzamido-3-phenylpropanamido)-3-phenylpropyl acetate, Figure 1). The ^1^H-NMR data of **1** compared favourably with those previously reported for saropeptide isolated from *Hypericum japonicum* [9], the roots of *Clausena anisata* [11] and *Zeyhera digitalis*[12] and stems of *Celastrus rugosus* [13].

Compound **2** was isolated as white crystals. Its molecular formular was determined as C_32_H_30_N_2_O_4_ based on ESI-MS peaks at m/z 506.22 [M+H]^+^ and 529 [M+Na]^+^. The ^1^H-NMR and ^1^H-^1^H-COSY spectra displayed two spin systems in the aliphatic region. The first began with a multiplet at *δ*_H_ 4.61, assigned to H-2 which was coupled with H-1b (4.52 m) and H-1a (4.01 dd (*J =* 4.3, 11.4) and also to H-3b at *δ*_H_ 2.98 dd (*J =* 6.4, 13.7) and H-3a at *δ*_H_ 2.87 dd (*J =* 8.4, 13.7). The second spin system began with a quartet at *δ*_H_ 4.90 (*J* = 6.7) assigned to H-2', which was coupled with H-3'b at *δ*_H_ 3.27 dd (*J =* 6.5, 13.9) and H-3'a at *δ*_H_ 3.19 dd (*J =* 7.1, 13.9). COSY correlations were also observed between the proton at *δ*_H_ 4.61 (1H, m, H-2) and an NH group at *δ*_H_ 6.64 (1H, d, *J* = 8.4, N-Hb), and the proton at *δ*_H_ 4.90 (1H, q, *J* = 6.7, H-2') and an NH group at *δ*_H_ 6.54 (1H, d, *J* = 6.5, N-Ha) (Figure 2). The aromatic region showed four sets of aromatic protons signals. These include two sets of 5H multiplets in two sets of AA'BB'C systems, which are compatible with four monosubstituted benzene rings. The analysis of the ^13^C-NMR and ^13^C-DEPT-135 showed the presence of two amide carbonyl functions *δ*_C_ 167.4 and 167.4 and an ester carbonyl function at *δ*_C_ 172.1, two benzylic methylene groups at *δ*_C_ (37.5 and 37.7) an oxymethylene (65.1), twenty two methine and four quarternary carbons. Compound **2** was thus elucidated as anabellamide (2-benzamido-3-phenylpropyl-2-benzamido-3-phenylpropanoate) (Figure 1) and the NMR data are consistent with those previously reported [9,10,11,14].

Compounds **1** and **2** were evaluated *in vitro* for trypanocidal activities against *T. b. rhodesiense*, *Trypanosoma cruzi* and for cytotoxicity against the mammalian L6 cells (Table 1).

**Table 1:** *In vitro* antitrypanosomal activity and cytotoxicity of dipeptides from *Zapoteca portoricensis* against *T.b rhodesiense T. cruzi* and cytotoxic effects against L6 cells.

Compound	*T. b. rhodesiense*	*T.cruzi*	L6 cells
	IC_50_ µM	IC_50_ µM	IC_50_ µM
**1**	3.63	41.65	92.05
**2**	12.21	16.05	71.20
Melarsoprol	0.003	-	-
Benznidazole	-	0.407	-
Podophyllotoxin	-	-	0.008

The IC_50_’s are mean values from at least two replicates (the variation is a maximum of 20%). The reference compounds and their IC_50_’s are shown.

The IC_50_ values against *T. b.rhodesiense* were 3.63 µM for **1** and 12.21 µM for **2**;while that against *T. cruzi* were 41.65 µM for **1** and 16.05 µM for **2**. The corresponding IC_50_ values for L6 cells were 92.05 µM and 71.20 µM, respectively. These results indicated strong activity of compound **1** against *T. b. rhodesiense* and moderate activity of compound **2** against *T. b. rhodesiense* and *T. cruzi*, with selectivity indices of 25.3, 4.4 and 5.8, respectively. In previous studies compound **1** has been found to possess anti-inflammatory activity as well as antiplatelet aggregation activity [15,16,17]. These properties are complementary to the observed trypanocidal property, since inflammation poses major problems, especially in the advanced stages of trypanosomiasis [18]. Platelet aggregation is also known to be induced by trypanosomiasis [19]. Compound **2** was found to possess an aromatase inhibitory activity [20]. Thus, these two compounds represents promising natural hits, especially compound **1** with a reasonably selectivity for *T. b. rhodesiense*.

## 3. Experimental

### 3.1. General Procedures

NMR spectra (^1^H, ^13^C, DEPT, HMQC and HMBC) were recorded with Bruker ARX 500 or AVANCE DMX 600 NMR spectrometers. MS (ESI) and HRMS (ESI) were obtained with Finnigan LCQ Deca and Maxis 4 G 20213 mass spectrometers, respectively. Analytical HPLC was carried out with a Dionex P580 HPLC system coupled to a photodiode array detector (UVD340S). Routine detection was at 235, 254, 280 and 354 nm. The separation column (125 × 4 mm, length × internal diameter) was prefilled with Eurospher-10 C18 (Knauer, Dusseldorf, Germany), and a linear gradient of nanopure water (adjusted to pH 2 by addition of formic acid) and methanol was used as eluent. Semipreparative HPLC was performed with a Merck Hitachi L-7100 system coupled to a UV detector (L-7400). A linear gradient of HPLC grade methanol and nanopure water was used in each case of separation.

### 3.2. Plant Materials

The roots of *Z. portoricensis* were collected in July, 2011 from Nsukka, in Enugu State, Nigeria. The plant was authenticated by Mr Alfred Ozioko, a taxonomist at the Centre for Ethnomedicine and Drugs Development, a subsidiary of the Bioresources and Development Center Programme (BDCP) in Nsukka, Enugu State, Nigeria. A voucher specimen of the root was deposited at the herbarium of the Department of Pharmacognosy, University of Nigeria, Nsukka, Enugu State, Nigeria with reference number Pcg/SN 89. The plant material was air dried at room temperature for about 2 weeks.

### 3.3. Exraction and Isolation

About 500 g of the dried powder of *Z. portoricensis* roots was extracted with methanol. The methanol extract (24.6 g) was dispersed in 200 mL of 10% aqueous methanol and successively extracted with hexane (500 mL × 3), ethyl acetate (500 mL × 3) and butanol (500 mL) to obtain hexane (HF), ethylacetate (EF) and butanol (BF) fractions, respectively. The ethyl acetate fraction was further separated by vacuum liquid chromatography on silica gel (230–400 mesh) eluting with gradients of hexane ethylacetate and then dichloromethane to obtain fractions F1 to 10. Fraction F4 was further separated on Sephadex LH-20 eluted with 50% of dichloromethane in methanol. The fractions were monitored on silica TLC plates developed with dichloromethane: methanol 9:1 and detection was done under UV 254 nm and by anisaldehyde spray. Fractions 20–69, which showed consistently similar spots were combined and finally purified by reverse phase semi-preparative HPLC to afford the two compounds **1** and **2**.

*Saropeptide* (**1**): Yield 2.0 mg; white crystals; ^1^H-NMR (500 MHz, CDCl_3_) δ: 7.69 d (2H, dd, *J =* 7.2 Hz, H-12/H-16), 7.50 (1H, t, *J =* 7.4, H-14), 7.42 (2H, t, *J =* 7.6, H-13/H-15), 7.20–7.28 (5H, m, H-5/H-6/H-7/H-8/H-9), 7.10–7.18 (3H, m, H-6'/H-7'/H-8'), 7.05 (2H, d, H-5'/H-9'), 6.72 (1H, d, *J =* 8.4, N-Hb), 5.89 (1H, d, *J =* 9.3, N-Ha), 4.74 (1H, q, H-2), 4.33 (1H, m, H-2'), 3.90 (1H, dd, *J =* 4.9, 11.3, H-1'b), 3.79 (1H, dd, *J =* 4.2, 11.3, H-1'a)), 3.19 (1H, dd, *J =* 5.9, 14.0, H-3b), 3.04 (1H, dd, *J =* 8.5, 14.0, H-3a), 2.73 (2H, t, *J =* 7.1, CH_2_-3'), 2.01 (3H, s, CH_3_-2''); MS (ESI+): *m/z* = 444.9 (55%) [M+H]^+^, 467(100%) [M+H]^+^, 403 (35%), 385 (15%).

*Anabellamide* (**2**): Yield 5.0 mg; white crystals; ^1^H-NMR (500 MHz, CDCl_3_) δ: 7.68 (2H, dd, *J* = 7.2 Hz, H-12'/H-16'), 7.64 (2H, dd, *J* = 7.2 Hz, H-12/H-16), 7.49 (1H, t, *J* = 7.4 Hz, H-14), 7.42 (1H, t, *J* = 7.4 Hz, H-14'), 7.37 (2H, t, *J* = 7.7 Hz, H-13/H-15), 7.29 (2H, overlap, H-13'/H-15'), 7.18–7.22 (5H, m, H-5/H-6/H-7/H-8/H-9), 7.26–7.29 (5H, m, H-5'/H-6'/H-7'/H-8'/H-9'), 6.64 (1H, d, *J* = 8.4 Hz, N-Hb), 6.54 (1H, d, *J* = 6.5 Hz, N-Ha), 4.90 (1H, q, *J* = 6.7 Hz, H-2'), 4.61 (1H, m, H-2), 4.52 (1H, m, H-1b), 4.01 (1H, dd, *J* = 11.4, 4.3 Hz, H-1a), 3.27 (1H, dd, *J* = 13.9, 6.5 Hz, H-3'b), 3.19 (1H, dd, *J* = 13.9, 7.1 Hz, H-3'a), 2.98 (1H, dd, *J* = 13.7, 6.4 Hz, H-3b), 2.87 (1H, dd, *J* = 13.7, 8.4 Hz, H-3a). ^13^C-NMR (125 MHz, CDCl_3_) δ: 172.1 (s, C-1'; ester C=O), 167.4 and 167.4 (both s, C-10 and C-10'; benzamide C=O), 137.4 (s, C-4), 136 (s, C-4'), 133.5 (s, C-11) 134.4 (s, C-11'), 132.3 (d, C-14), 131.6 (d, C-14'), 129.5 (d, C-5/C-9), 129.1 (d, C-5'/C-9'), 128.9 (d, C-13/C-15), 128.8 (d, C-13'/C-15'), 129.4 (d, C-6'/C-8'), 128.7 (d, C-6/C-8), 127.6 (d, C-7'), 127.4 (d, C-12'/C-16'), 127.3 (d, C-12/C-16), 127 (d, C-7), 65.6 (t, C-1), 54.7 (d, C-2'), 50.5 (d, C-2), 37.7 (t, C-3'), 37.5 (t, C-3). MS (ESI+): *m/z* = 506 (30%) [M+H]^+^, 529 (52%) [M+Na]^+^, 239 (100%); 292 (30%).

### 3.4. Determination of in Vitro Antitrypanosomal Activity and Cytotoxicity

Minimum essential medium (50 µL) supplemented according to a standard method [21], with 2-mercaptoethanol and 15% heat inactivated horse serum was added to each well of a 96-well microtiter plate. Serial drug dilutions were prepared covering a range from 90 to 0.123 µg/mL. Then 10^4^ bloodstream forms of *T. b. rhodesiense* STIB 900 in 50 µL culture medium were added to each well and the plate incubated at 37 °C under a 5% CO_2_ atmosphere for 72 h. Ten microlitres of Alamar Blue (12.5 mg resazurin dissolved in 100 mL distilled water) were then added to each well and incubation continued for 2–4 h. The plate was then read in a Spectramax Gemini XS microplate fluorometer (Molecular Devices Cooperation, Sunnyvale, CA, USA) using an excitation wavelength of 536 nm and emission wavelength of 588 nm. Fluorescence development was measured and expressed as percentage of the control. Data were transferred into the graphic programme Softmax Pro (Molecular Devices) which calculated IC_50_ values [22]. Melarsoprol was used as standard drug.

Cytotoxicity was determined using rat skeletal myoblast (L6) cells. The culture medium was RPMI 1640 supplemented with L-glutamine 2 mM, HEPES 5.95 g/L, NaHCO_3_ 2 g/L and 10% foetal bovine serum. Podophyllotoxin (Sigma-Aldrich) was used as the reference drug. The assay was performed following the antitrypanosomal assay protocol [21]. The IC_50_ values were calculated from the sigmoidal inhibition curves using Softmax Pro software (Molecular Devices). Tests were done in three independent experiments in duplicate.

## 4. Conclusions

The two dipeptides saropeptide (**1**) and anabellamide (**2**) were isolated for the first time from *Z. portoricensis*. Investigation of their trypanocidal activity as well as their cytotoxicity using rat skeletal myoblast (L6) cells showed that compound **1** exhibited potent activity against *T. b.rhodesiense* with high selectivity index, while compound **2** exhibited moderate activities against *T. b.rhodesiense* and *T. cruzi*.

## References

[1] Nwodo N.J., Uzochukwu C.I. (2008). Studies on anti-inflammatory and antimicrobial activities of crude methanol extracts of *Zapoteca portoricensis* Jacq. H. Hernanadez. Recent Prog. Med. Plants.

[2] Nwodo N.J., Omeje E.O., Brun R. (2009). *In vitro–in vivo* studies on anti-trypanosomal potentials of *Zapoteca portoricensis*. Asian Pac. J. Trop. Med..

[3] Agbo M.O., Okoye F.B.C., Nwodo N.J. (2010). *In vivo* anti-inflammatory effect of *Zapoteca portoricensis* (Jacq) HM Hernández. Inter. J. Health Res..

[4] Esimone C.O., Onuh P.U., Obitte N.C., Egege M.K., Ugoeze K.C. (2009). *In vitro* evaluation of lozenges containing extracts of roots of *Zapoteca portoricensis* (FAM: Fabaceae). J. Pharmacol. Toxicol..

[5] Ukwe C.V., Ubaka C.M., Adibe M.O., Okonkwo C.J., Akah P.A. (2010). Antiulcer activity of roots of *Zapoteca portoricensis* (Fam. Fabaceae). J. Basic Clin. Pharm..

[6] Agbafor K.N., Akubugwu E.I., Ogbashi N.E., Ajah P.M., Ukwandu C.C. (2011). Chemical and Antimicrobial properties of leaf extracts of *Zapoteca portoricensis*. Res. J. Med. Plants.

[7] Danquah M.K., Agyei D. (2012). Pharmaceutical applications of bioactive peptides. OA Biotechnol..

[8] Hartmann R., Meisel H. (2007). Food-derived peptides with biological activity: From research to food applications. Curr. Opin. Biotechnol..

[9] Ishiguro K., Nagata S., Fukumoto H., Yamaki M., Takagi S., Isoi S. (1991). A dipeptide derivative from *Hypericum japonicum*. Phytochemistry.

[10] Hashim N.M., Rahmani M., Shamaun S.S., Ee G.C.L., Sukari M.A., Ali A.M., Go R. (2011). Dipeptide and xanthones from *Artocarpus kemando* Miq. J. Med. Plant Res..

[11] Kouam J.L., Dongo E., Mpondo T.N., White R.L. (2012). Chemical constituents from stem bark and roots of *Clausena anisata*. Molecules.

[12] Ferreira D.T., Silva R.B., Deoliveira A.B., Isobe M., Braz R. (1995). Dipeptide from the root of *Zeyhera digitalis*. J. Braz. Chem. Soc..

[13] Chang R., Wang C., Zeng Q., Guan B., Zhang W., Jin H. (2013). Chemical constituents of the stems of *Celastrus rugosus*. Arch. Pharm. Res..

[14] Carvalho M.G., Cardozo M.A., Catunda F.E., Carvalho A.G. (2010). Chemical constituents of *Piptadenia gonoacantha* J.F. Macbr. Ann. Braz. Acad. Sci..

[15] Kuo P.C., Hwang T.L., Lin Y.T., Kuo Y.C., Leu Y.L. (2011). Chemical constituents from *Lobelia chinensis* and their anti-virus and ant-inflammatory bioactivities. Arch. Pharm. Res..

[16] Catalan C.A.N., de Heluani C.S., Kotowicz C., Gedris T.E., Herz W. (2003). A linear sesterterpene, two squalene derivative and two peptide derivatives from *Croton hieronymi*. Phytochemistry.

[17] Wu T.S., Chan Y.Y., Liou M.J., Lin F.W., Shi L.S., Chen K.T. (1998). Platelet aggregation inhibitor from *Murraya euchrestifolia*. Phytother. Res..

[18] Lundkvist G.B., Sellix M.T., Nygard M., Davis E., Straume M., Kristensson K., Block G.D. (2010). Clock gene expression during chronic inflammation induced by infection with *Trypanosoma brucei brucei* in rats. J. Biol. Rhythms..

[19] Nwagwu M., Inyang A.L., Molokwu R.I., Essen E.M. (1989). Platelet aggregating activity of released factor(s) from *Trypanosoma brucei brucei*. Afr. J. Med. Sci..

[20] Balunas M.J., Su B., Riswan S., Fong H.H.S., Brueggemeier R.W., Pezzuto J.M., Kinghorn A.D. (2009). Isolation and characterisation of aromatase inhibitor from *Brassaiopsis glomerulata* (Araliaceae). Phytochem. Lett..

[21] Baltz T., Baltz D., Giroud C., Crockett J. (1985). Cultivation in a semi-defined medium of animal infective forms of *Trypanosoma brucei, T. equiperdum, T. evansi, T. rhodesiense* and *T. gambiense*. EMBO J..

[22] Orhan I., Sener B., Kaiser M., Brun R., Tasdemir D. (2010). Inhibitory activity of marine sponge-derived natural products against parasitic protozoa. Mar. Drugs.

